# Investment Behavior Related to Automated Machines and Biased Technical Change: Based on Evidence From Listed Manufacturing Companies in China

**DOI:** 10.3389/fpsyg.2022.874820

**Published:** 2022-04-27

**Authors:** Hong Jiang, Xue Wang, Qian Xiao, Silin Li

**Affiliations:** ^1^College of Business, Shanghai University of Finance and Economics, Shanghai, China; ^2^College of Business, Wenzhou University, Wenzhou, China

**Keywords:** automated machines, technical change bias, ordinary capital, labor structure effect, RAMOC

## Abstract

This paper studies the impact of a recent increase in the ratio of automated machines to ordinary capital (RAMOC) on the bias of technical change in the manufacturing industry and the mechanism influencing this. Using panel data of A-share listed manufacturing companies on the Shanghai and Shenzhen stock exchanges from 2012 to 2019, combined with the Xtfrontier model and *trans*-log production function, we measure the index of the bias of technical change of the manufacturing industry in China. Furthermore, we adopt a fixed effects model to test the impact of an increase of investment in automated machines on the bias of technical change. We also use an intermediary effect model to examine the intermediate mechanism from the perspectives of capital and skill matching. The results show that technical change in the manufacturing industry is biased toward automated machine capital. An incremental increase in RAMOC leads to technical change in the manufacturing industry becoming biased toward automated machine capital, wherein the intermediary mechanism is the labor structure effect. Based on industrial linkage, the investment in automated machines in the upstream (downstream) manufacturing industry increases, the technical change of the downstream (upstream) manufacturing industry is biased toward automated machine capital, and the forward linkage effect is greater than the backward linkage effect. This research enhances understanding of (1) the direction and characteristics of technical change in China, (2) how to improve the output efficiency of automated machines, (3) differences in factor revenue distribution, and (4) how new growth points in the economy can be cultivated. They show that we should encourage and support investment in automated machines, vigorously promote technical change to bias toward automated machine capital, improve the skill level of the labor force, and strengthen the match between automated machines and labor.

## Introduction

In recent years, artificial intelligence and digital technologies have led to a technological revolution and industrial change (Blake and Frajtova, 2021; Cohen and Macek, 2021; Galbraith and Podhorska, 2021). Moreover, investment in automated mechanisms has rapidly increased. In 1993, industrial robots numbered only 79,135 million worldwide; however, this increased to 403,519 million by 2019. The overall ownership of industrial robots in the world has also gradually increased. In 2016, China became the country with the largest inventory of robots, accounting for 19% of the total global inventory^1^. This paper focuses on how the strategic decision by companies to increase investment in automated machines in the context of digital technologies and artificial intelligence affects the bias of technical change. Previous research has mainly focused on two aspects of the bias of technical change: (1) whether technical change is biased toward labor or investment, which is used to explain changes in the pricing and income distribution of these two factors; and (2) whether technical change is biased toward skilled or unskilled labor. These studies have found that the skill premium in the United States at the end of the 20th century was generated by skill-biased technical change (Acemoglu, 2002; Song et al., 2010; Wang et al., 2014).

However, while previous studies have classified labor into skilled and unskilled, which helps to measure the bias of technical change, few papers have studied the investment measures of technical change bias. In the critical times of countries experiencing the changes in their development mode, economic structure, and growth momentum, there is an urgent need to examine the effect of automated machines on technical change and economic growth, given trends of a greater aging population, rising labor costs, and normalization of the COVID-19 epidemic. The role of technical change is not isolated, but is coupled with various factors of production that could be elaborated by technical change bias (Li and Zhu, 2016). Technical change is biased toward a certain factor, indicating that it helps to increase the marginal output of this factor (Hicks, 1932), which further affects the structure of economic growth and the distribution of factor income (Lu and Zhang, 2013). Therefore, with the increased global investment in automated machines, special attention should be paid to whether technical change is biased toward automated machine capital and the examination of the coupling relationship between technical change and automated machines. In order to increase the promotion effect of automated machines on economic growth and enhance the core competitiveness of enterprises, it is also necessary to study the mechanisms influencing the strategic decision to invest in automated machines and the bias of technical change.

This paper studies whether technical change is biased toward automated machines, and the mechanism influencing this. It does so by dividing capital into automated machines and ordinary capital and examining the ratio between these [ratio of automated machines to ordinary capital (RAMOC)]. The results show that the technical change of the manufacturing industry is biased toward automated machines. Particularly, if RAMOC increases by 1%, the automated-machine-biased technical change index increases by 0.03%. The results of the mechanism analysis and mediation effect examination show that as RAMOC increases, the ratio of two types of labor input that match the two types of capital increases (yielding a labor structure effect). This increases the marginal output of automated machines and biases technical change toward automated machines. Heterogeneity analysis shows that the increase of automated machine investment in upstream and downstream industries significantly contributes to the bias of technical change toward automated machines, and reveals that the forward linkage effect is greater than the backward linkage effect. The relative increase of investment in automated machines by non-state-owned manufacturing enterprises contributes to this bias, while the effect on state-owned manufacturing enterprises is not significant. The increase of investment in automated machines by high-tech manufacturing enterprises contributes more to technical change bias compared with increases in low-technology industries. Understanding the stage and heterogeneity characteristics of China’s current technological progress is significant for investigating the output efficiency of automated machines, explaining the difference in income distribution factors and finding new impetus for economic growth.

Our contributions can be elaborated as follows. (1) We enrich the understanding of not only biased technical change, but also the direction of technical change in China’s manufacturing industry. Based on studies by DeCanio (2016) and Aum et al. (2018), we divide capital into automated machine and ordinary capital. Findings show that the technical change of the manufacturing industry is biased toward automated machines, combined with the continuous increase in automated machine investment. Drawing on the definition of skill-biased technical change proposed by Acemoglu (2002), this paper defines automated-machine-biased technical change as technical change that promotes an increase in the ratio of the marginal output of automated machines to ordinary capital.

(2) This study enriches the discussion of mechanisms influencing the bias of technical change. Previous literature has explored the role of market scale effect, price effect, international trade, government policy, and other factors on the bias of technical change. The study also deeply analyzes how the intermediate influencing mechanism—the increase in investment in automated machines (the market scale effect)—affects the bias of technical change. From the perspective of matching capital and labor skills, we examine the premise that if RAMOC increases enterprises that correspondingly invest in more production factors related to investment in automated machines to fully implement the improvement effect of technical change on the investment productivity of automated machines. Moreover, with cost constraints, enterprises will reduce their input of other production factors that match ordinary capital. Hence, they will increase the marginal output of automated machine investment to facilitate the bias of technical change toward automated machines. In addition, from the perspective of industrial linkage and the technology spillover effect, we verify that an increase in RAMOC in the upstream (downstream) manufacturing industry makes the technical change of the downstream (upstream) manufacturing industry biased toward automated machines, providing a reference for improving the marginal output of automated manufacturing machines. (3) Previous empirical research has used samples related to biased technical change based mostly on regional and industry data, with large sampling granularity. This paper provides evidence at the enterprise level, as micro-data, can capture the heterogeneity characteristics of the bias of technical change in different types of manufacturing enterprises. Hence, we further study the differential impact of the bias of technical change of various manufacturing enterprises on the relative increase in investment in automated machines and provide an accurate and reliable basis for policy formulation.

The remainder of this article is arranged as follows. The next section comprises the literature review and hypotheses. This is followed by details of the research design and the empirical results. Subsequently, we provide a discussion and conclude by summarizing our findings and the implications of our research.

## Literature Review and Hypotheses

This paper explores the factors influencing the bias of technical change. Hicks (1932) stated that technical change is biased toward production factors that have higher prices. Therefore, the price of automated machines and ordinary capital will affect the bias of technical change. Inspired by Hicks, Habakkuk (1962) found that the scarcer the production factors, the higher the price. Hence, technical change tends to be a scarcer factor. Therefore, the input scale of automated machines and ordinary capital factors will influence the price of these two factors and further influence the bias of technical change. Kennedy (1964) did not focus on the influencing factors relative to price, and believed that technical change bias depends on the factor related to saving effect. Technical change helps to reduce the inputs of production factors, labor, and investment. There is a relationship between the economics of these two factors—that is, the innovation possibility boundary—which can constrain the bias of technical change. Acemoglu (2002) added micro-enterprise subjects and incorporated technology monopoly manufacturers into a theoretical model on the basis of previous research. The author suggested that technology monopoly manufacturers produce equipment drawn from the principle of profit maximization. The bias of technical change depends on two factors: price effect and market scale effect. Price effect refers to the fact that technology monopoly manufacturers have greater power to develop and adopt technologies that can produce more expensive products, while market scale effect refers to the fact that technology monopoly manufacturers have greater power to develop and adopt certain technologies so that their products can have a larger market scale. The dominant effect depends on the substitution elasticity between the two factors: if the substitution elasticity is greater than 1, the market scale effect plays a leading role; if the substitution elasticity is less than 1, the price effect plays a leading role; finally, if the substitution elasticity is equal to 1, technical change is neutral. In fact, no matter which effect dominates, with the increase of the inputs of production factor *X* relative to another production factor *Y*, enterprises will invest more production factors related to factor *X* to enable the full improvement effect of technical change on the productivity of abundant factor *X*. Furthermore, under cost constraints, enterprises need to reduce the production factors’ inputs related to factor *Y*. Therefore, the marginal output of factor *X* will increase and bias the technical change toward factor *X*.

Many scholars have used this theory to explain why technical change in the United States has been biased toward skilled labor, as input into skilled labor increased in the second half of the 20th century (Acemoglu, 2002; Krusell et al., 2003; Weiss and Garloff, 2011). According to Acemoglu (2002), with the increase of one type of capital input relative to that of another, technical change is biased toward the capital in which there has been greater investment, regardless of whether the substitution elasticity of automated machines and ordinary capital is greater than or less than 1 (i.e., whether the market size effect or the price effect dominates).

The intermediate transmission mechanism of the change of the relative input scale of automated machines and ordinary capital on the bias of technical change comprises the relative input of the two types of labor force matching the two types of capital. With an increase in RAMOC, enterprises will invest more in production factors that match the investment in automated machines (e.g., high-skilled laborers who undertake more complex and high-level tasks) to enable the full facilitating effect of technical change on automated machines’ productivity. Furthermore, under cost constraints, enterprises will reduce the input of production factors that match ordinary capital (e.g., low-skilled labor related to ordinary capital) to increase the marginal output of automated machines, facilitating the progress of technology with a bias toward automated machines. In particular, the most typical representative of automated machine capital is industrial robots. Some researchers (Autor et al., 2003; Acemoglu and Restrepo, 2019) have stated that if investment in automated machines increases, machines are more likely to replace low-level, highly repetitive, and relatively simple tasks (mainly undertaken by low-skilled labor) to obtain more cost and productivity advantages, resulting in the substitution effect of investment in automated machines on low-skilled labor. For example, industrial robots initially mainly replaced low-skilled labor, including handling, transmission, welding, and laser processing. Although automation technology rapidly developed, the evaluation of systems and management was slower, and education lagged behind. As a result, the substitution effect of automated machines on low-skilled labor with lower education increased with the surge in investment in automated machines (Deng and Huang, 2019). At the same time, with the increase in investment in automated machines, more new and high-level complex tasks were created, and these are not easily replaced by machines. Thus, labor has a comparative advantage, meaning that the demand for highly skilled labor that matches the abilities of automated machines will increase (Autor et al., 2003; Acemoglu and Restrepo, 2018). In turn, high-skilled jobs related to automation R&D (Research and Development) design, equipment manufacturing and applications could increase. For example, every 10 large intelligent robots need an artificial intelligence engineer, while small robots need more engineers (Deng and Huang, 2019). Currently, there are nearly 1.5 million intelligent robots in use, equating to 150,000 artificial intelligence engineers (Deng and Huang, 2019). Automated machine capital can undertake tasks that humans are not qualified, or are unwilling, to carry out, such as those that exceed the limits of human sensory and cognitive abilities (e.g., precision instruments). Robots can also work in an environment in which humans cannot be accommodated (e.g., deep space exploration), or where there are high risks or great mental intensity. This results in a creative effect on highly skilled labor (Deng and Huang, 2019). Therefore, if RAMOC increases, automated machines will replace the low-skilled labor force that matches ordinary capital, and this will create high-skilled labor that matches automated machines. That is, the proportion of high- and low-skilled labor input increases as RAMOC increases. When this occurs, the productivity of automated machines could also increase due to the labor structure effect, biasing technical change toward automated machine (Bessen, 2018; Stevenson, 2018). Based on the above analysis, we propose the following hypothesis:

*Hypothesis 1 (H1): The increasing ratio of automated machines to ordinary capital has a positive impact on automated-machine-biased technical change.*

Enterprise ownership (i.e., state-owned vs. non-state-owned) will affect investment behavior and decisions about technical change (Marshall, 1907). Therefore, it will also impact the effect of RAMOC on the bias of technical change. Due to the significant difference in the labor structure effect between state-owned enterprises and non-state-owned enterprises, the impact of RAMOC on the bias of technical change is heterogeneous. A core task of government lies in ensuring the stability of the labor force and high-quality employment. This is of great significance for the development of a country’s economy and the harmony and stability of society. The size of the labor force in China is increasing in recent years, reaching 811 million in 2019^2^. The employment pressure is also formidable in China, especially in the face of the complex international environment and recent domestic reform and development tasks^3^. State-owned enterprises have a responsibility to bear a large number of redundant and low-skilled employees to achieve the government’s employment goals, provide stable employment, and secure the social benefits of employment (Dong and Putterman, 2003). Therefore, the effect between the increase in investment in automated machines and the bias toward them in state-owned enterprises is not significant (Dong and Putterman, 2003).

There is a solid political relationship between state-owned enterprises and the government in China. State-owned enterprises are able choose to enter an industry that deviates from their existing factor endowment structure in the case of no “self-viability.” This is why most state-owned enterprises are distributed across capital- and energy-intensive industries, such as oil, coal, and metallurgy. State-owned enterprises also face problems arising from a redundant and low-skilled labor force, resulting in lower production efficiency and poorer operating performance compared with non-state-owned enterprises, which inhibits the demand for high-skilled labor (Li and Guo, 2015). However, non-state-owned enterprises have a relatively weak political relationship with the government. This can improve their competitiveness and resource allocation efficiency in situations with relatively few restrictions on decision-making. This can ensure their survival and sustainable development, reducing the burden of a great number of redundant or low-skilled employees (Gong et al., 2015). With investment in automated machines increasing, non-state-owned enterprises will correspondingly invest more in high-skilled labor that matches the abilities of automated machines to improve productivity, and will reduce low-skilled labor that matches ordinary capital under cost constraints. This can increase the marginal output of automated machines, significantly biasing technical change toward automated machines. Based on the above analysis, the following hypothesis is proposed:

*Hypothesis 2 (H2): A relative increase of automated machine investment of non-state-owned manufacturing enterprises contributes to the automated machine bias of technical change, while the effect of state-owned manufacturing enterprises is not significant.*

The investment behavior regarding automated machines varies across enterprises. Technical change is triggered by resource endowments and motivations (Hsu et al., 2014). As a result, RAMOC of enterprises with different types of technology has a heterogeneous effect on the bias of technical change. Compared with low-tech industries, high-tech industries are capital intensive and technology intensive. They have higher technical barriers for entry, focusing on R&D and innovation. In high-tech industries, enterprises have high production efficiency, a wider extent of technology, and added value, so enterprises have a stronger motivation to invest in automated machines and promote technical change (Dobrzanski et al., 2021). When the investment in automated machines increases, the creation effect of high-tech industries on high-skilled labor also rises. Low-tech industries are generally labor intensive, relying on the cost advantages of labor to obtain economic benefits. However, although low-tech industry enterprises also have an incentive to invest in automated machines because of high labor costs, the marginal output of automated machines is low. This is because production in low-tech industries is characterized as low-skilled and does not use many high-tech automated machines. Therefore, we propose that:

*Hypothesis 3 (H3): An increase of automated machine investment in high-tech manufacturing enterprises contributes more to the automated-machine-biased technical change compared to that of low-technology industries.*

In addition to enterprise ownership and technology type, the effect of RAMOC on technical change bias caused by the correlation degree of upstream and downstream industries is worthy of attention. These industries have an input–output relationship which manifests as an industrial linkage effect or a mutually related production network. Input–output theory is important for studying the degree of industrial linkage (Leontief, 1936). Production factor inputs and technical advances in the upstream (downstream) could influence those in the downstream (upstream) through industrial linkage effects (Kesavayuth and Zikos, 2012). Industrial linkage can be divided into forward and backward. Forward linkage refers to an industry’s links with its forward industry partners with respect to production factor inputs and technical extent, while backward linkage refers to an industry’s links with its backward industry with respect to production factor inputs and technical extent. The degree of influence and scope of the industrial linkage depend on the nature of the industry and its position in the production network. For example, the forward linkage effect of an upstream industry (e.g., the raw materials industry) on a downstream industry is wide and deep, and the downstream industry (e.g., the final consumer goods production sector) has a relatively low backward linkage effect on the upstream industry (Rui et al., 2010).

Based on H1 and the industrial linkage effect, a relative increase in investment in automated machines in upstream (downstream) manufacturing enterprises can facilitate the bias of technical change toward automated machines. Because upstream and downstream industries have a certain linkage effect based on factor supply, final consumer goods sales, technological innovation, the production efficiency, and the automated technology level of the downstream (upstream) industry are further improved so that downstream (upstream) enterprises will correspondingly input more high-skilled labor that are related to automated machines and reduce the input of low-skilled labor that are related to ordinary capital. This will increase the marginal output of downstream (upstream) automated machines and promote the technical change of downstream (upstream) manufacturing industries to bias toward automated machines. Moreover, products of upstream manufacturing enterprises are widely adopted and more malleable than downstream ones, and downstream enterprises have more intermediate investment from the upstream, which results in the downstream being more affected by the upstream. Hence, the downstream needs to tightly link with the technical change of upstream enterprises (Zhang et al., 2020). Therefore, the promotion effect of increasing RAMOC in upstream enterprises on the automated-machine-biased technical change in downstream enterprises (forward linkage effect) is greater than the positive effect of the ratio of two types of investments in downstream enterprises on this technical change in upstream enterprises (backward linkage effect). Therefore, it is theoretically expected that:

*Hypothesis 4 (H4): An increase of automated machine investment in upstream and downstream industries significantly contributes to automated-machine-biased technical change, wherein the forward linkage effect is greater than the backward linkage effect.*

## Research Design

### Sample Selection and Data Sources

In this research, A-share listed manufacturing companies on the Shanghai and Shenzhen stock exchanges in 2012–2019 were selected as subjects. The sample selection was based on a serious shortage of data on automation and ordinary capital in 1990–2011 and the lack of labor force matching investment in automated machines and ordinary capital in 2020. Additionally, data on Special Treatment (ST) and Star Special Treatment (*ST) companies were excluded along with were data on listed companies that were suspended, delisted or abeyant, and abnormal data. Since outliers may affect empirical results, we trimmed all variables at the 1st and 99th percentiles. The data on listed manufacturing companies were obtained from the Choice database, and the data on the manufacturing industry were obtained from the China Statistical Yearbook^4^ and the National Bureau of Statistics of China.^5^

### Analytical Techniques

To solve the problem of missing variables triggered by the change of time and individuals, we used a dual fixed effects model that controlled for individuals and time in the baseline regression. In particular, we measured technical change bias on the basis of an Xtfrontier model and trans-log production function. There are three main methods for measuring the direction of technical change: the constant elasticity of substitution production function method, the normalized supply-side system approach, and the *trans*-log production function. The *trans*-log production function could slacken the condition that technical change is neutral and scale-rewarded, making it suitable for research on the bias of technical change of multi-factor inputs (Christensen et al., 1973; Zhang et al., 2015). To solve the problem of endogeneity, we controlled the interaction terms of time, year, and industry, which dealt with the problem of missing variables. We also adopted Bartik’s instrument to address the bidirectional causality problem referred to by Paul et al. (2020) and Zhao et al. (2021). Bartik’s instrumental variables are popular among researchers (Paul et al., 2020; Zhao et al., 2021). The basic idea entails simulating estimates of the calendar year with the initial share composition of the variables and the overall growth rate, which are highly correlated with the actual values but are not related to other residual terms. For robustness, we used the placebo test method. Although we addressed the endogeneity problem, identification of the causal relationship between RAMOC and technical change bias may also entail other challenges. As labor costs and the aging population are increasing and the information technology industry is rapidly developing, technical change in manufacturing may tend to be biased toward automated machines. In terms of mechanism testing, we used the widely adopted mediation effect model referred to by Baron and Kenny (1986). We used STATA software to estimate all models.

### Regression Model and Variable Definitions

To verify the proposed hypotheses, we established the basic model as follows:


(1) $$bias_{it}=\beta_{0}+\beta_{1}m_{it}+\gamma X+u_{i}+v_{t}+\epsilon_{it}$$

where *bias*_*it*_ is the dependent variable which represents the value of the manufacturing technical change bias index measured by the translog production function. Furthermore, *bias*_*it*_ reflects the direction and extent of the impact of technical change on the relative marginal output of the two types of investment (automated machines and ordinary capital). In particular, *bias*_*it*_ > 0 denotes that technical change makes the marginal output growth rate of automated machine capital greater than that of ordinary capital, which means that the direction of technical change is considered to be biased toward automated machines. *bias*_*it*_ < 0 means that the technical change is biased toward ordinary capital. *bias*_*it*_ = 0 shows that technical change is neutral (Diamond, 1965; Khanna, 2001). The calculation of *bias*_*it*_ was performed as follows.

First, the trans-log production function is:


(2) $$\begin{array}{c} lnY_{it}=\beta_{0}+a_{1}lnM_{it}+a_{2}lnK_{it}+a_{3}lnL_{it}+a_{4}Z_{it}\\ \;+a_{5}t0.5a_{6}{\left(lnM_{it}\right)}^{2}+0.5a_{7}{\left(lnK_{it}\right)}^{2}+0.5a_{8}{\left(lnL_{it}\right)}^{2}\\ \;+0.5a_{9}{\left(lnZ_{it}\right)}^{2}+0.5a_{10}t^{2}+a_{11}lnM_{it}lnK_{it}+a_{12}lnM_{it}lnL_{it}\\ \;+a_{13}lnK_{it}lnL_{it}+a_{14}lnM_{it}lnZ_{it}+a_{15}lnK_{it}lnZ_{it}\\ \;+a_{16}lnL_{it}lnZ_{it}+a_{17}tlnM_{it}+a_{18}tlnK_{it}+a_{19}tlnL_{it}\\ \;+a_{20}tlnZ_{it}+v_{it}-u_{it} \end{array}$$

where *Y*_*it*_ represents the total output of each manufacturing firm using the operation revenue of listed manufacturing firms (Giannetti et al., 2015). *M*_*it*_ and *K*_*it*_ represent the stock of automated machines and ordinary capital input, respectively. Considering data availability, automated machine capital uses data on the net value of the machinery and electronic equipment in listed manufacturing firms. The data on net value of other fixed assets were utilized as ordinary capital (DeCanio, 2016; Aum et al., 2018).

Furthermore, *Z*_*it*_ and *L*_*it*_ indicate high-skilled and low-skilled labor input, respectively, where high-skilled labor represents the R&D and technical workforce. The other workforces are denoted as low-skilled labor (Shen, 2016). Furthermore, *t* (*t* = 1, 2, …) is the time trend which denotes technical change. β_0_ denotes the mean of the cross-section effect. *a*_1_, *a*_2_, *a*_3_, *a*_4_, and *a*_5_ represent the accumulation effect of production factors, namely, automated machines, ordinary capital, low-skilled labor inputs, high-skilled labor inputs, and technical change, respectively. *a*_6_, *a*_7_, *a*_8_, *a*_9_, and *a*_10_ represent the scale effect of production factors. *a*_11_, *a*_12_, *a*_13_, *a*_14_, *a*_15_, *a*_16_, *a*_17_, *a*_18_, *a*_19_, and *a*_20_^6^ represent the coordination effect between the two production factors. *v*_*it*_ represents the random error of firm *i* in *t* time period. *u*_*it*_ represents technical inefficiencies in the production process of firm *i* in *t* time period, *u*_*it*_≥0, which measures the gap between actual output and technical frontier output.

According to equation (2), the output elasticity of the elements *M*_*it*_ and *K*_*it*_ are as follows:


(3) $$\begin{array}{c} \varepsilon_{M}=\partial lnY_{it}/\partial lnM_{it}=a_{1}+a_{6}lnM_{it}+a_{11}lnK_{it}\\ \;+a_{12}lnL_{it}+a_{14}lnZ_{it}+a_{17}t \end{array}$$

(4) $$\begin{array}{c} \varepsilon_{K}=\partial lnY_{it}/\partial lnK_{it}=a_{2}+a_{7}lnK_{it}+a_{11}lnM_{it}\\ \;+a_{13}lnL_{it}+a_{15}lnZ_{it}+a_{18}t \end{array}$$

Furthermore, the marginal output of automated machine *M*_*it*_ and ordinary capital *K*_*it*_ could be expressed as:


(5) $$\begin{array}{c} MP_{M_{it}}=\varepsilon_{M}\times \frac{Y_{it}}{M_{it}}=\frac{Y_{it}}{M_{it}}\\ \;\times \left(a_{1}+a_{6}lnM_{it}+a_{11}lnK_{it}+a_{12}lnL_{it}+a_{14}lnZ_{it}+a_{17}t\right) \end{array}$$

(6) $$\begin{array}{c} MP_{K_{it}}=\varepsilon_{K}\times \frac{Y_{it}}{K_{it}}=\frac{Y_{it}}{K_{it}}\\ \;\times \left(a_{2}+a_{7}lnK_{it}+a_{11}lnM_{it}+a_{13}lnL_{it}+a_{15}lnZ_{it}+a_{18}t\right) \end{array}$$

Finally, the technical change bias index is computed based on Diamond (1965) and Khanna (2001):


(7) $$bias_{it}=\frac{\partial MP_{M_{it}}/\partial t}{MP_{M_{it}}}-\frac{\partial MP_{K_{it}}/\partial t}{MP_{K_{it}}}=\frac{a_{17}}{\varepsilon_{M}}-\frac{a_{18}}{\varepsilon_{K}}$$

In equation (1), *m*_*it*_ is the main explanatory variable of equation (1), *m*_*it*_ = *M*_*it*_/*K*_*it*_, which represents the ratio of automated machines to ordinary capital. *X* is the control variable, which includes enterprise export scale (*Xlnex*_*it*_), marketing concentration (*hhi*_*it*_), total factor productivity (*tfp*_*it*_), enterprise age (*lnage*_*it*_), and average wage of employees (*lnw*_*it*_) (Song et al., 2010; Wang et al., 2014). The six control variables above are used to control the enterprise’s overseas demand, industry competition, enterprise production efficiency and innovation ability, enterprise life cycle, and the impact of labor costs on *bias*_*it*_. In addition, *u_i_*, *v_t_* represent fixed effects of individual and time, respectively.^7^ Lastly, *ϵ*_*it*_ is the random error. All the variables above in equation (1) are set out in Table 1.

**TABLE 1:** Variable definitions.

Types	Symbols	Name	Definition
Dependent variable	*bias*_*it*_	Biased technical change index	The direction and extent of the impact of technical change on the relative marginal output of the two kinds of capital (automated machines and ordinary capital).
Independent variables	*m*_*it*_	The ratio of automated machines to ordinary capital investment (RAMOC)	*m*_*it*_=*M*_*it*_/*K*_*it*_
	*lnex*_*it*_	The export scale	Natural logarithm of operating revenue in Hong Kong, Macao, Taiwan, and foreign countries.
	*hhi*_*it*_	The marketing concentration	The Herfindahl index of the two-digit manufacturing industry, measured as the sum of the squares of an industry’s share of total industry revenues.
	*tfp*_*it*_	The total factor productivity	Using the OP (Olley and Pakes) method, the enterprise’s operating income performs as the output variable. The substantial transfer of the enterprise’s actual controller performs as the enterprise withdrawal. The net fixed assets perform as the capital stock. The cash paid for fixed assets, intangible assets, and other long-term assets performs as the actual investment amount. The total number of employees performs as the labor input variable. The cash paid for goods and labor performs as an intermediate input variable.
	*lnage*_*it*_	The enterprise age	Log (current year – registration year +1).
	*lnw*_*it*_	The average wage of employees	Natural logarithm of average wage of employees in listed manufacturing enterprises.

## Empirical Results

### Descriptive Analysis

**FIGURE 1:**
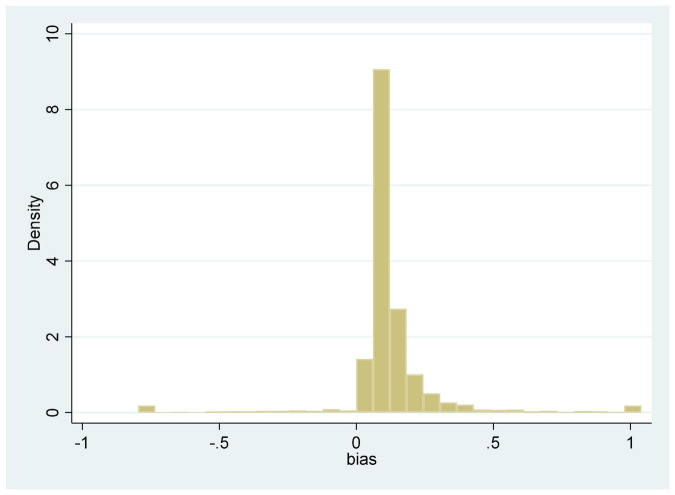
Histogram of technical change bias index of Chinese listed manufacturing enterprises from 2012 to 2019.

Table 2 shows the descriptive statistics of the main variables. The results for the manufacturing technical change bias index in 2012–2019 were obtained by calculating equation (7) using the Xtfrontier model in STATA software, shown in Figure 1. From our observation of the results in Table 2 and Figure 1, we find that the value of *bias* is generally greater than 0 and the mean is 0.1431, demonstrating that manufacturing technical change is biased toward automated machine capital in China. In addition, the full distance of *bias* is large and the standard deviation is 0.2321, indicating that there are obvious differences among different manufacturing enterprises in the technical change bias index. The mean and standard deviation of *m* are 1.0806 and 1.3390, respectively, demonstrating that investment in automated machines in manufacturing is larger than ordinary capital and that there are large differences among different manufacturing enterprises in the two types of investment. It can be seen that only micro-data can accurately capture the effect of the relative increase of investment in automated machines on the bias of technical change.

**TABLE 2:** Descriptive analysis.

Variable	*N*	Mean	Median	*SD*	Min	Max	Skewness	Kurtosis
*bias*	2749	0.1431	0.1133	0.2321	–0.9894	1.4377	0.9551	19.9064
*m*	2749	1.0806	0.6796	1.3390	0.0514	9.5420	3.8409	21.8810
*lnex*	2749	18.9047	19.1689	2.2391	6.0768	25.4970	–0.6815	4.2113
*HHI*	2749	0.0495	0.0378	0.0414	0.0139	0.2284	2.0934	7.9625
*TFP*	2749	7.1942	7.1625	0.3775	6.4088	8.2567	0.4333	2.9707
*lnage*	2749	2.6786	2.7726	0.4792	0	4.1589	–1.5154	7.6768
*lnw*	2749	11.4639	11.4167	0.4470	5.0024	14.6230	0.3228	9.1225

Table 3 shows the Pearson correlation coefficient of the main variables. The results show that automated machine investment (*m*) has a significantly positive effect on the bias of technical change (*bias*), with a significance level of 5%. The correlation coefficient between every two variables is less than 0.5, implying that the problem of collinearity is not serious.

**TABLE 3:** Correlation analysis.

*bias*	*m*	*lnex*	*hhi*	*tfp*	*lnage*	*lnw*	
*m*	1						
*lnex*	0.0338**	1					
*HHI*	–0.3112***	0.0197	1				
*TFP*	–0.1097***	–0.0508***	–0.0021	1			
*lnage*	–0.1484***	–0.0406***	0.2581***	–0.0495***	1		
*lnw*	–0.0362**	–0.1203***	0.1705***	–0.0484***	0.3787***	1	
*bias*	0.1537***	–0.0027	0.0558***	–0.1138***	0.2762***	–0.0376***	1

***, **, refer to significance at 1 and 5%, respectively.

### Hypothesis Testing

#### Results of Principal Regression and Analysis

Table 4 shows the results that RAMOC has a positive impact on the automated-machine-biased technical change t based on equation (1) and the related theoretical analysis. This supports H1.

**TABLE 4:** Results of principal regression.

Variables	*bias*
*M*	0.0290***
	(2.7685)
*lnex*	–0.0178***
	(–5.3533)
*HHI*	–0.4295**
	(–2.1419)
*TFP*	–0.0131
	(–0.7372)
*lnage*	0.0029
	(0.0468)
*lnw*	0.0409**
	(2.2239)
*N*	2749
adj. *R*^2^	0.6539
Enterprises fixed effect	Control
Years fixed effect	Control

Numbers in the brackets are t-values, standard errors are clustered at the enterprise level, ***, **, refer to significance at 1 and 5%, respectively.

#### Heterogeneity Analysis

The bias of technical change toward automated machines varies across different types of manufacturing enterprises, as elaborated in Figure 1, making it necessary to conduct heterogeneity tests. Considering the differences of typical ownership and industry in production factors’ input and innovation development in China, we analyzed heterogeneity from two aspects, as follows:

(1) Heterogeneity analysis of ownership. The listed manufacturing companies can be divided into two types: state-owned and non-state-owned.^8^ The regression results grouped according to ownership are elaborated in columns (1) and (2) of Table 5, indicating that the relative increase of investment in automated machines of non-state-owned manufacturing enterprises contributes to the automated-machine-biased technical change, while, for the state-owned manufacturing enterprises, this is not significant, supporting H2.

**TABLE 5:** Heterogeneity analysis.

Variables	(1)	(2)	(3)	(4)	(5)
	State-owned	Non-state-owned	High-tech	Medium-high-tech	Low-tech
	*z_bias*	*Z_bias*	*z_bias*	*z_bias*	*z_bias*
*Z_m*	–0.0010	0.2447***	0.3217***	0.0702*	0.0526*
	(–0.0177)	(4.0222)	(3.9320)	(1.8351)	(1.9144)
*z_lnex*	–0.2920***	–0.1973***	–0.3246***	–0.1768***	–0.1036***
	(–2.8614)	(–5.2495)	(–4.1774)	(–4.1237)	(–3.7847)
*z_HHI*	–0.1384	–0.0716	–0.6693**	0.0633	–0.0605***
	(–1.6150)	(–1.5532)	(–2.1802)	(0.5170)	(–2.8803)
*z_TFP*	–0.1820	0.0116	–0.0738	0.0126	0.0283
	(–2.0124)	(0.3643)	(–1.1388)	(0.2984)	(1.0526)
*z_lnage*	–0.2895	–0.0042	–0.0116	0.1675	–0.2981***
	(–1.1493)	(–0.0251)	(–0.0403)	(0.7010)	(–2.7537)
*z_lnw*	0.1408**	0.0927*	0.1536**	0.1048	0.0025
	(2.5378)	(1.8672)	(2.0961)	(1.5579)	(0.1069)
*N*	665	2084	1086	977	686
*adj. R^2^*	0.7447	0.6292	0.6187	0.7065	0.6892
Enterprises fixed effect	Control	Control	Control	Control	Control
Years fixed effect	Control	Control	Control	Control	Control

Numbers in the brackets are t-values, standard errors are clustered at the enterprise level, ***, **, * refer to significance at 1, 5, and 10%, respectively.

(2) Heterogeneity analysis of the industry. Referring to the Organization for Economic Cooperation and Development (OECD) manufacturing technology classification standard in 2011 and the Industrial Classification for National Economic Activities of China in 2011 and 2017, this research divided the industries of listed manufacturing companies into three types: high-tech, medium-high-tech, and low-tech enterprises.^9^ The grouped regression results are shown in columns (3) to (5) of Table 5, indicating that the relative increase of investment in automated machines by high-tech manufacturing enterprises contributes more to the automated-machine-biased technical change compared with low-technology industries, supporting H3.

#### Industry Linkage Effect Test

Considering the correction effect among different industries, we examined the effect not only in the manufacturing industry, but also in its upstream (downstream) industries. We modeled the ratio of two types of investment in the upstream (*forward*_*m*_*it*_) and downstream (*forward*_*m*_*it*_), respectively, which can be expressed as follows (Lu et al., 2017; Zhu et al., 2020):


(8) $$forward\_m_{ijt}=\sum_{j\neq s}\left(input_{jst}/\sum_{s}input_{jst}\right)\times m_{ijt}$$

(9) $$backward\_m_{ijt}=\sum_{j\neq x}\left(output_{jxt}/\sum_{x}output_{jxt}\right)\times m_{ijt}$$

where *input*_*jst*_ represents the intermediate products that industry *j* obtained from upstream industry *s*, and the total intermediate products from all upstream industries are ∑_*s*_*input*_*jst*_. *output*_*jxt*_ represents the intermediate products that industry *j* sold to downstream industry *x*, and the total intermediate products sold to downstream industries are ∑_*x*_*output*_*jst*_. The data of *input*_*jst*_ and *output*_*jxt*_ can be obtained from the Input–Output Table of China in 2012, 2017, and 2018.^10^ We used these three years to calculate the direct consumption and distribution coefficient in the span of 2012 to 2019 (Zhu et al., 2020).^11^ We reformed equation (1) by replacing the main explanatory variable *m*_*it*_ with the variables *forward*_*m*_*ijt*_ and *backward*_*m*_*ijt*_, and examined the effect of the ratio of two types of investment in the manufacturing upstream (or downstream) industry. The regression results are provided in Table 6. The results show that the coefficients of *forward*_*m* (0.04) and *backward*_*m* (0.01) are significantly positive, which supports H4. This indicates that the relative increase of automated machine investment in upstream and downstream industries significantly contributes to the automated-machine-biased technical change, and that the forward linkage effect is greater than the backward linkage effect.

**TABLE 6:** Industry association effect test.

Variables	(1)	(2)
	*bias*	*bias*
*forward_m*	0.0395**	
	(2.4701)	
*backward_m*	–0.0178***	0.0058*
	(–5.3338)	(1.9196)
*lnex*	–0.0178***	–0.0176***
	(–5.3338)	(–5.4283)
*HHI*	–0.4930**	–0.4359**
	(–2.3950)	(–2.1267)
*TFP*	–0.0170	–0.0139
	(–0.9625)	(–0.7758)
*lnage*	–0.0055	–0.0079
	(–0.0892)	(–0.1277)
*lnw*	0.0423**	0.0409**
	(2.2848)	(2.1887)
*N*	2749	2749
*R* ^2^	0.7272	0.7222
Enterprises fixed effect	Control	Control
Years fixed effect	Control	Control

Numbers in the brackets are t-values, standard errors are clustered at the enterprise level, ***, **, * refer to significance at 1, 5, and 10%, respectively.

### Robustness Test

#### Replacement of Proxy Variables

To remeasure the technical change bias index of manufacturing (*bias*_1_*it*_), we replaced the high-skilled labor by employees with a bachelor’s degree or above and replaced the low-skilled labor by employees with a bachelor’s degree or less (Wang et al., 2014). The results are elaborated in column (1) of Table 7. The coefficient of automated machine investment (*m*_*it*_) is still significantly positive. Hence, the conclusion of this research is solid.

**TABLE 7:** Robustness test.

Variables	(1) Replace proxy variables	(2) Sub-sample regression 2012-2016	(3) Sub-sample regression 2017-2019	(4) Placebo test
	*bias*__1_	*bias*	*bias*	*bias*__2_
*m*	0.0024*	0.0153***	0.0383***	–0.0010
	(1.7340)	(2.8955)	(3.4819)	(–0.8031)
*lnex*	–0.0026***	–0.0205***	–0.0060	0.0001
	(–2.7368)	(–3.6617)	(–1.1307)	(0.2406)
*HHI*	–0.0988*	–0.1007	0.3757	–0.0327
	(–1.7435)	(–0.4757)	(0.5233)	(–0.3857)
*TFP*	–0.0031	–0.0211*	–0.0077	0.0001
	(–0.7561)	(–1.8820)	(–0.1597)	(0.0740)
*lnage*	–0.0210	–0.0393	0.3357	–0.0089
	(–0.9378)	(–0.4575)	(1.3566)	(–0.4099)
*lnw*	0.0008	0.0161	0.0134	0.0002
	(0.2530)	(1.2767)	(0.2917)	(0.1125)
*N*	3307	1209	1380	563
adj. *R*^2^	0.8009	0.7964	0.6613	0.8173
Enterprises fixed effect	Control	Control	Control	Control
Years fixed effect	Control	Control	Control	Control

Numbers in the brackets are t-values, standard errors are clustered at the enterprise level, ***, **, * refer to significance at 1, 5, and 10%, respectively.

#### Sub-Sample Regression

As the originating year of artificial intelligence was identified as 2017 (Ye, 2017), this study used 2017 as the boundary year, leading to division of the sample into 2012–2016 and 2017–2019. Hence, the regression results are elaborated in columns (2) and (3) of Table 7. The coefficient of the main explanatory variable is still significantly positive, and the coefficients in 2017–2019 are significantly greater than the values before 2017. As a result, manufacturing enterprises paid more attention to the enhancement of automation and artificial intelligence technology from the original year of artificial intelligence technology. In addition, they increased investment in automated machines.

#### Placebo Test

To prevent the impact of other unknown factors on the selection of experiment units, we adopted a placebo test. In the 21st century, the technical change of manufacturing enterprises may have a trend of automated machine bias with the rapid development of global information, the Internet, and automation technology, coupled with the emergence of artificial intelligence technology. Considering this situation, the estimation above may confuse the effect of increasing RAMOC on improving the level of technical change with the previous trend of automated-machine-biased technical change in manufacturing. To solve this problem, we examined the relationship between automated-machine-biased technical change of manufacturing enterprises before 2012 and the increase of investment in automated machines through a placebo test. Using the RAMOC of the period of 2012–2019, we estimated the effect on the bias of technical change (*bias*_2*m*) in 2004–2011. The regression results are shown in column (4) of Table 7. We found that the coefficient of the main explanatory variable is not significant, which ensures the robustness of the benchmark regression results.

### Endogeneity Control

#### Control of Fixed-Effect Interaction Terms

Based on equation (1), this study further controlled fixed-effect interaction terms, including the intersection of enterprises and years, the intersection of enterprises and industry, and the intersection of years and industry to solve the endogenous problem caused by missing variables. Column (1) in Table 8 shows the regression results. The effect on *bias*remains significantly positive at the 1% level of controlling fixed-effect interaction terms, which means that a trivial endogenous problem caused by missing variables exists.

**TABLE 8:** Endogenous analysis.

Variables	(1)	(2)	(3)
		First stage	Second stage
	*Bias*	*m*	*bias*
*H_iv*		0.1427***	
		(20.5451)	
*m*	0.0291***		0.0794***
	(2.7636)		(10.6245)
*lnex*	–0.0180***	0.0059	–0.0193***
	(–5.3540)	(0.3254)	(–5.7809)
*HHI*	–0.3159*	3.1137*	–0.4467
	(–1.6523)	(1.7789)	(–1.3062)
*TFP*	–0.0122	–0.0305	–0.0249
	(–0.6768)	(–0.3458)	(–1.4837)
*lnage*	–0.0012	–0.3759***	0.0560
	(–0.0186)	(–2.6873)	(0.8422)
*lnw*	0.0414**	–0.0234	0.0416***
	(2.2797)	(–0.3511)	(3.1150)
*N*	2749	2940	2243
adj. *R*^2^	0.6494	0.8053	0.3450
Control the intersection of enterprises and years	Control		
Control the intersection of enterprises	Control		
Control the intersection of years and industry	Control	Control	Control
Enterprises fixed effect	Control	Control	Control
Years fixed effect		79.8236	15.5733

Numbers in the brackets are t-values, standard errors are clustered at the enterprise level, ***, **, * refer to significance at 1, 5, and 10%, respectively.

#### Bartik Instrument

The RAMOC in manufacturing enterprises and the automated-machine-biased technical change may exhibit reverse causality. To settle this problem, we used the share movement method to construct a Bartik instrument (Paul et al., 2020; Zhao et al., 2021). We redefined automated machine investment *M*_*ijt*_, where *j* two-digit industry investment is composed of multiple enterprises’ investment and the set of *j* is expressed as *J*. We can conclude the equation relationship as:


(10) $$M_{jt}=\sum_{i\epsilon J}M_{ijt}\;K_{jt}=\sum_{i\epsilon J}K_{ijt}$$

(11) $$H_{jt}=M_{jt}/K_{jt}\;H_{ijt}=M_{ijt}/K_{ijt}$$

where *t* = *t*_0_, *t*_0_ is the initial year (2012 in this study), *M*_*ijt*_0__ is the automated machine investment of enterprise *i* in industry *j* for the initial year *t*_0_, *K*_*ijt*_0__ is the ordinary capital of enterprise *i* in industry *j* for the initial year *t*_0_, and *g*_*it*_ is the growth rate in year *t* relative to the initial year *t*_0_ for the automated machine and ordinary capital input.^12^ The Bartik instrument *H*_*iv*_*ijt*_ can be expressed as follows:


(12) $$H\_iv_{ijt}=\sum_{i\epsilon J}M_{ijt_{0}}\times \left(1+g_{it}\right)/\sum_{i\epsilon J}K_{ijt_{0}}\times \left(1+g_{it}\right)$$

A Bartik instrument (*H*_*iv*_*ijt*_) is an instrumental variable of *H*_*ijt*_ obtained by *M*_*ijt*_0__, *K*_*ijt*_0__, and *g*_*it*_, which is obviously highly correlated to *m*_*it*_ in equation (1). If properly controlling the fixed effects at the individual, industry, and year levels, the Bartik instrument will not be correlated with other residual terms affecting *bias*_*ijt*_. Therefore, *H*_*iv*_*ijt*_ meets the basic requirements of instrumental variables. Columns (2) and (3) of Table 8 report the results in the first and second stages, respectively. This indicates that the estimated value of *m*_*it*_ using the share movement method is highly correlated with the actual valuation, and that the main explanatory variables are significantly positive at the 1% level. Consequently, our conclusion is solid.

### Further Testing: Mechanism Analysis

As discussed above, we found that an increase in RAMOC will promote the bias of technical change toward automated machines. Next, through analyzing the previous related theory, we further facilitate the discussion of the reasons of this effect. We found that an increase in RAMOC could increase the ratio of labor input matching automated machines to other labor input matching ordinary capital. Hence, there was a technical change bias toward automated machines. The mediation effect can be modeled (Baron and Kenny, 1986) as follows:


(13) $$bias_{it}=\beta_{0}+\beta_{1}m_{it}+\gamma X+u_{i}+v_{t}+\epsilon_{it}$$

(14) $$lnsk_{it}=\beta_{0}+\beta_{1}m_{it}+\gamma X+u_{i}+v_{t}+\epsilon_{it}$$

(15) $$bias_{it}=\beta_{0}+\beta_{1}m_{it}+\beta_{2}p_{it}+\gamma X+u_{i}+v_{t}+\epsilon_{it}$$

where *lnsk*_*it*_ is an intermediary variable, *sk*_*it*_ is the labor structure denoting the ratio of high-skilled labor force and low-skilled labor force. The other variables’ definitions are consistent with the benchmark model, equation (1). The regression results are shown in Table 9.

**TABLE 9:** The results of mechanism analysis.

Variables	(1)	(2)	(3)
	*bias*	*lnsk*	*bias*
*m*	0.0225***	0.0113*	0.0220***
	(3.7354)	(1.8429)	(3.7092)
*lnex*	–0.0170***	–0.0146	–0.0164***
	(–5.4009)	(–1.2215)	(–5.2776)
*HHI*	–0.3583**	–0.1708	–0.3505**
	(–2.0279)	(–0.1469)	(–2.0010)
*TFP*	–0.0173	–0.0564	–0.0147
	(–0.9799)	(–1.0451)	(–0.8370)
*lnage*	0.0048	0.3942	–0.0132
	(0.0778)	(1.4502)	(–0.2146)
*lnw*	0.0413**	0.2097***	0.0317*
	(2.2934)	(3.4050)	(1.7635)
*lnsk*			0.0456***
			(6.0503)
*N*	2749	2749	2749
adj. *R*^2^	0.6676	0.8170	0.6742
Enterprises fixed effect	Control	Control	Control
Years fixed effect	Control	Control	Control

Numbers in the brackets are t-values, standard errors are clustered at the enterprise level, ***, **, * refer to significance at 1, 5, and 10%, respectively.

The coefficients of *m*_*it*_ and *lnsk*_*it*_ are significant, which meets the requirements of the mediation mechanism test. *m*_*it*_ has a positive impact on *lnsk*_*it*_, indicating that the proportion of high-skilled labor force increases with investment in automated machines. The coefficient *lnsk*_*it*_ is significantly positive in column (3), which means that the increase of the ratio of high-skilled labor to low-skilled labor could promote the technical change to bias toward automated machines. This is because high-skilled labor highly matches automated machines, and it could help to increase the marginal output of automated machines so that technical change is biased toward automated machines. As a result, an increase in RAMOC promotes technical change to bias toward automated machines by increasing the ratio of high-skilled to low-skilled labor inputs.

## Discussion

This study was conducted using technical change bias theory and industrial linkage theory based on the increasing investment in automated machines and combined with the transcendent logarithmic production function and use of the Xtfrontier model to measure the technical change bias index in China’s manufacturing industry. Drawing on data of listed enterprises in China’s manufacturing industry from 2012 to 2019, we find that manufacturing technical change is biased toward automated machines in China. Furthermore, an increase in RAMOC affects the ratio of two types of labor inputs matching the two types of investment to further influence the bias of technical change.

First, an increase in RAMOC will promote technical change with bias toward automated machines. In Table 4, the regression results of the main explanatory variable *m* shows that the coefficient is 0.0290 and significant at the level of 1%, indicating that an increase in RAMOC will promote technical change with bias toward automated machines. The results are consistent with those of Acemoglu (2002), Weiss and Garloff (2011), Acemoglu and Restrepo (2019), and Cette et al. (2021). On one hand, the enterprises’ investment behavior tends to match the production factors related to automated machines. On the other hand, it is necessary to reduce the other production factors’ input that matches ordinary capital based on the cost constraints of enterprises, so that the productivity and marginal output of automated machines can be improved. Therefore, technical change is biased toward automated machines.

In recent years, automated machines have begun to perform more intelligently as artificial intelligence technology flourishes globally and becomes deeply integrated with diversified industries. This trend will create a highly skilled workforce to match the development of automation, such as employees engaged in automation R&D, design, equipment manufacturing, and applications. Moreover, low-skilled labor related to ordinary capital, such as those engaged in repetitive, simple, and low-level tasks, will be replaced. The match between the investment in automated machines and labor force further improves the marginal output of automated machines to promote technical change to bias toward automated machines.

Second, the increase of investment in automated machines by non-state-owned manufacturing enterprises contributes to automated-machine-biased technical change, while the effect in state-owned manufacturing enterprises is not significant. In columns (1) and (2) of Table 5, the impact of *m* on *bias* is significantly positive for non-state-owned manufacturing enterprises, while it is not significant for state-owned manufacturing enterprises. This is because state-owned manufacturing enterprises carry the burden of employing redundant and low-skilled labor to stabilize and ensure employment. This leads to a substitution effect in which a low-skilled labor force matched with ordinary capital is not significant for increasing automated machine investment (Dong and Putterman, 2003; Chang et al., 2019). Meanwhile, the misallocation of resources in state-owned enterprises is more serious, and production efficiency is low, which can impair creative vitality in automated technology. Therefore, the technical change of state-owned enterprises is not significantly biased toward automated machine capital. Moreover, as results show, the means of RAMOC in non-state-owned and state-owned enterprises are 1.29 and 1, respectively. The reason for this is that in order to improve production efficiency and pursue high profits and core competitiveness, there is no serious employment redundancy and low-skilled labor employment in non-state-owned enterprises. These enterprises have greater motivation to invest in automated machines, which has a replacement effect on low-level, simple, and repetitive jobs. This creates employment that matches the abilities of automated machine capital and improves the marginal output of the automated machines, resulting in technical change being significantly biased toward investment in automated machines.

Third, compared with low-tech industries, the relative increase of automated machine investment of high-tech manufacturing enterprises contributes more to the automated-machine-biased technical change. In columns (3) to (5) of Table 5, the impact of *m* on *bias* is significantly positive for high-tech, medium-high-tech, and low-tech industries, and the coefficients are 0.3217, 0.0702, and 0.0526, respectively. This shows that when comparing the effect of an increase in RAMOC on promoting technical change to bias toward automated machines for different types of industries, the high-tech industry has the greatest effect, followed by the medium-high-tech industry and the low-tech industry. This can be explained by the fact that high-tech and medium-high-tech industries are capital- and technology-intensive. They focus on high technology and R&D, while low-tech industries are labor intensive. The mean RAMOC of high-tech, medium-high-tech, and low-tech industries is 1.54, 1.25, and 1.08, respectively. Therefore, compared with medium-high-tech industries and low-tech industries, the high-tech industries prefer to invest much more in automated machines. Meanwhile, the match between more advanced automation technology and high-skilled labor is complementary with automated machines. The productivity of automated machines in high-tech industries is relatively high, and the product margins of automated machine investment is large. As a result, the relative increase of investment in automated machines of high-tech manufacturing enterprises contributes more to automated-machine-biased technical change for the high-tech industry. In addition, for low-tech industries, RAMOC is the smallest, product technology and added value are lowest, and the high-skilled labor is least matched with automated machines, which leads to the product margins of its automated machines being least. Hence, the effect of RAMOC on the bias of technical change is smallest in low-tech industries.

Fourth, the relative increase of automated machine investment can significantly contribute to the automated-machine-biased technical change in both upstream and downstream industries. Furthermore, the forward linkage effect is greater than the backward linkage effect. In Table 6, the investment in automated machines of the upstream (downstream) manufacturing industry can promote technical change to be biased toward automated machines in the downstream (upstream). On the one hand, derived from the technical spillover effects, the bias toward automated machines in the upstream (downstream) triggered by the investment in automated machines could facilitate technical change to bias toward automated machines in the downstream (upstream). On the other hand, the increment of relative input of automated machine investment in upstream (downstream) manufacturing industries could promote the labor skill level and the proportion of the entire manufacturing industry to match the investment in automated machines, increasing the marginal output of automated manufacturing and biasing technical change toward automated machines for the downstream (upstream) industry. Interestingly, the coefficient of *forward*_*m* (.0395) is larger than that of *backward*_*m* (.0098), indicating that the impact of the increase in investment in automated machines of upstream enterprises on the technical change of downstream enterprises (forward linkage effect) is greater than the impact of the increase in downstream enterprises’ investment in automated machines on the technical change of upstream enterprises (backward linkage effect). This is because there are more intermediate inputs in the downstream industry, which gives the upstream industry deep, wide influence on the downstream industry. Thus, downstream industries need more capital, labor, and technology to match those of upstream industries (Zhang et al., 2020).

## Results

### Conclusion

This article studied the effect of an increase in RAMOC on the bias of technical change in manufacturing. The results can be summarized as follows. (1) In manufacturing, technical change is biased toward automated machines in China. (2) Compared with ordinary capital, greater investment in automated machines could bias technical change toward automated machines. The intermediate impact mechanism is the labor structure effect; that is, increasing the ratio of two types of labor force, respectively matching automated machines and ordinary capital. (3) Heterogeneity analysis results show that, first, RAMOC for non-state-owned manufacturing enterprises will significantly promote automated-machine-biased technical change, while state-owned manufacturing enterprises are not significant. Second, in comparing the effect of an increase in RAMOC on promoting the bias of technical change toward automated machines for different types of industries, the high-tech industry showed the greatest effect, followed by the medium-high-tech and the low-tech industries. (4) The relative increase of automated machine investment can significantly contribute to automated machine-biased technical change in both upstream and downstream industries, and the forward linkage effect is greater than the backward linkage effect.

The findings are particularly relevant to theory and practice regarding investment behavior in manufacturing. First, they enrich understanding of the bias of technical change and the direction of technical change of the manufacturing industry in China. Considering that investment in automated machines continues to increase rapidly, and focusing on the relationship between technical change and automated machine investment, this study divided capital into automated machines and ordinary capital. Measurements showed that the marginal output growth rate of automated investment is greater than that of ordinary capital. Therefore, we found that the technical change in the manufacturing industry is biased toward automated machines.

Second, this study explored the mechanisms that are influential on the bias of technical change from a new perspective. We explored how technical change can be biased toward automated machines with an increase of investment therein from the perspective of matching capital and labor skills, and industry linkage. The results show that when RAMOC increases, the ratio of labor force input matching the two types of capital also increases, which promotes technical change bias toward automated machines. With respect to upstream and downstream industries, the study showed that an increase of investment in automated machines in both upstream and downstream industries can significantly contribute to biasing technical change toward automation.

Third, the study enriches empirical research on the bias of technical change by examining how a relative increase of investment in automated machines affects the bias of technical change in manufacturing using a dual fixed effects model and micro-data. Furthermore, it adopted an intermediary effect model to estimate the intermediate mechanism.

### Implications

This study has several policy implications. First, to encourage and support investment into automated machines and vigorously promote automated-machine-biased technical change, the Chinese government should implement policy tools; fiscal and taxation measures; and credit, finance, and insurance initiatives. It should also increase R&D support for automated machines, promote leading enterprises and academic institutions to undertake technical research and achieve transformation in related fields, improve the marginal output of investment in automated machines, and cultivate new growth points for China’s economy.

Second, the study suggests developing a differentiated automated machine investment and technical change strategy according to the enterprise ownership and technology type. It is necessary to fully enable the effect of automated machine investment in non-state-owned and high-tech industries, and to enhance the effectiveness of investment in automated machines in state-owned and low-tech industries. According to the current situation in relation to the bias of technical change across enterprises, differentiated automated machine investment and automation technology development strategies should be formulated.

Third, in the process of promoting technical change to bias toward the automation of manufacturing enterprises, the linkage effect between industries plays a role. It is necessary to pay attention to the impact of investment in automated machines for manufacturing on the bias of technical change in upstream and downstream industries. To promote the coordinated three-dimensional development of technical change in different industries, the industrial linkage effects of upstream and downstream industries could be used to help technical change develop a bias toward automated machines.

Furthermore, it is necessary to improve the skill level of the labor force and strengthen the match between automated machine investment and labor skills. This study shows that a relative increase in investment in automated machines will promote technical change that is biased toward them. The main channel therein is the match of automated machine investment and labor skills, since the higher the degree of matching, the greater the promotion effect. Therefore, with a continuous increase in investment in automated machines and a continuous development of automation technology, there will be a demand for high-skilled labor that matches the abilities of automated machines. Thus, this part of the labor force will be required to develop higher-skill levels. Meanwhile, there will be a substitution effect on the low-skilled labor force that engages in simple, repetitive, and low-level tasks. Therefore, it is necessary to continuously improve the skill levels of the high-skilled labor force so that they match those of automated machines. This can be undertaken through enterprises, governments, or third-party agencies for high-quality and targeted vocational skills training. The government can implement subsidies in skills, tax incentives, and other measures to incentivize transformation of the low-skilled labor force into a high-skilled labor force. In addition, it should strengthen training of the labor force in relation to automated machines through school and social education, and increase the market supply of this type of labor force.

### Research Limitations and Future Research Directions

Although this paper presents some valuable conclusions and makes several contributions to theoretical and empirical research, it is subject to several limitations. First, due to data constraints, we only used representative unlisted companies in the manufacturing industry, and the results differ from those using data on unlisted companies in the manufacturing industry. Future research should investigate the status of investment in automated machines and technical advances in unlisted enterprises, and further verify the causal relationship between RAMOC and technical change bias. Second, the factors influencing the bias of technical change mainly include price effects and market scale effects (Acemoglu, 2002). However, again due to data limitations, this paper only focused on the market scale effect and did not discuss price effects. This would require the price data of automated machines and ordinary capital. Therefore, as data are enriched, further examination regarding the impact of price effects will be possible.

## Data Availability Statement

The original contributions presented in the study are included in the article/supplementary material, further inquiries can be directed to the corresponding author/s.

## Author Contributions

HJ determined the research theme, research framework, and data analysis method, and was responsible for the finalization of the manuscript. XW was responsible for literature collation and draft writing. QX was responsible for writing the research hypotheses, data analysis, and discussion. SL was responsible for writing the empirical analysis. All authors contributed to the article and approved the submitted version.

## Conflict of Interest

The authors declare that the research was conducted in the absence of any commercial or financial relationships that could be construed as a potential conflict of interest.

## Publisher’s Note

All claims expressed in this article are solely those of the authors and do not necessarily represent those of their affiliated organizations, or those of the publisher, the editors and the reviewers. Any product that may be evaluated in this article, or claim that may be made by its manufacturer, is not guaranteed or endorsed by the publisher.

## Footnotes

1 Data source is international federation of robotics (IFR) https://ifr.org/.

2 Data source is National Bureau of Statistics of China. http://www.stats.gov.cn/.

3 The reference is Report on the Work of the Government of China in 2021.

4
http://www.stats.gov.cn/tjsj/ndsj/

5
https://data.stats.gov.cn/

6 Where *a*_11_ denotes the coordination effect between automated machines and ordinary capital; *a*_12_ denotes the coordination effect between automated machines and low-skilled labor; *a*_13_ denotes the coordination effect between ordinary capital and low-skilled labor; *a*_14_ denotes the coordination effect between automated machines and high-skilled labor; *a*_15_ denotes the coordination effect between ordinary capital and high-skilled labor; *a*_16_ denotes the coordination effect between low-skilled labor and high-skilled labor; *a*_17_ denotes the coordination effect between automated machines and technical change; *a*_18_ denotes the coordination effect between ordinary capital and technical change; *a*_19_ denotes the coordination effect between low-skilled labor and technical change; and *a*_20_ denotes the coordination effect between high-skilled labor and technical change. If the value of these coefficients is positive, it means that the two production factors have a complementary effect, while if the value is negative, it means that they have a substitute effect.

7 We used an *F* test and Hausman test, and the results rejected the mixed model regression and random effect estimation. In addition, by conducting the joint significance test of annual dummy variables, the *p*-value of the *F* test equaled to 0.0000, rejecting the null hypothesis of “no time effect.” Moreover, the dual fixed effects model of individual and time addressed the problem of missing variables changing with time and individual variation.

8 In order to compare the samples of different ownership enterprises directly, we needed to standardize all variables before conducting the group regression and apply the same treatment for the other group regressions.

9 High-tech industries include pharmaceutical manufacturing, special equipment manufacturing, computer, communication and other electronic equipment manufacturing, and instrumentation manufacturing. Medium-high-tech industries include chemical raw materials and chemical products manufacturing, general equipment manufacturing, automobile manufacturing, railways, ships, aerospace and other transport equipment manufacturing, electrical machinery, and equipment manufacturing. Low-tech industries include petroleum, coal and other fuel processors, rubber and plastic products, non-metallic mineral products, ferrous metal smelting and rolling processors, non-ferrous metal smelting and rolling processors, metal products enterprises, machinery and equipment repairs, agricultural and sideline food processors, food manufacturers, wine, beverages, and refined tea manufacturers, businesses involved in tobacco products, textiles and garments, clothing, leather, fur, feathers and their products, footwear, wood processing and wood, bamboo, rattan, grass products, furniture manufacturing, paper and paper products, printing and recording media reproduction, art, culture and education, sports and recreational products manufacturing, chemical fiber manufacturing, waste resources, and comprehensive utilities.

10 Data taken from the official website of the National Bureau of Statistics of China (https://data.stats.gov.cn/).

11 The direct consumption and distribution coefficients in the spans of 2012–2014, 2015–2017, and 2018–2019 were replaced by 2012, 2017, and 2018, respectively.

12 To calculate *g*_*it*_, we used the data of growth rate of two-digit industry-level fixed assets in year *t* relative to the initial year *t*_0_. The data of fixed assets is from the Statistical Yearbook of China from 2012–2019 (http://www.stats.gov.cn/tjsj./ndsj/).
